# Experiencing, Regulating, and Expressing Emotions: Gendered and Agentic Pathways of Emotional Labor in Human Services

**DOI:** 10.3390/bs15091245

**Published:** 2025-09-12

**Authors:** Yean Wang, Shuge Xu, Guanghuai Zheng

**Affiliations:** 1 School of Government, Beijing Normal University, Beijing 100875, China; echowang@bnu.edu.cn (Y.W.); xushuge@mail.bnu.edu.cn (S.X.); 2 School of Sociology, Nankai University, 38 Tongyan Road, Tianjin 300350, China

**Keywords:** emotional labor, gender, professional efficacy, work meaningfulness, humanistic management, human services

## Abstract

The expansion of emotional labor into the human services sector has raised concerns about emotional exhaustion and gender inequality, yet the potential for emotional work to serve as a source of motivation and meaning remains underexplored. This study reconceptualizes emotional labor as a dynamic, agentic process encompassing three stages: experiencing, regulating, and expressing emotion. Drawing on a nationally representative, multi-source dataset from the first round of the China Social Work Longitudinal Study (N = 5965), we examine how this emotional process unfolds differently by gender and how professional efficacy mediates the relationship between role ambiguity and work meaningfulness. We further explore how organizational governance—specifically internal governance and governmental support—moderates these relationships. Our findings reveal that women demonstrate stronger emotional regulation and derive greater work meaningfulness through professional efficacy, particularly under low governmental support, whereas men’s emotion processes are more direct and enhanced by organizational governance. These results underscore the importance of gender-sensitive and organizationally informed approaches to managing emotional labor. By integrating gendered agency and institutional context, this study offers a new framework for understanding emotional work and vocational development in the human services sector.

## 1. Introduction

Emotional labor is both essential and invisible in the human services sector (Sloan, 2014; Wharton, 2009). Social workers, nurses, and counselors are expected to manage their feelings with composure and care, even in emotionally charged, ambiguous, and under-resourced environments (Delgado et al., 2017). These expectations are rarely matched by adequate recognition, compensation, or organizational support (Grandey et al., 2015; Krstić et al., 2025; Nguyen & Stinglhamber, 2020). Researchers have investigated the effects of emotional labor on career outcomes. They have found that emotional labor is related to job dissatisfaction, professional burnout, depersonalization, work–family imbalance, and other unfavorable outcomes (Grandey et al., 2015; Tang & Gu, 2024). Because of the inherent paradox between inauthentic emotion and the actual self, displaying unfelt emotions may help to meet professional and organizational demands in the short term but, over time, can undermine emotional well-being and career longevity. Consequently, emotional labor has been questioned in terms of whether emotion should be commodified and how it affects career sustainability (Smith, 2014; Wharton, 2009). As was introduced in Hochschild’s (1983) groundbreaking book, the emotional requirements of workers are facing remonstration in the workplace and career governance.

**Figure 1:**
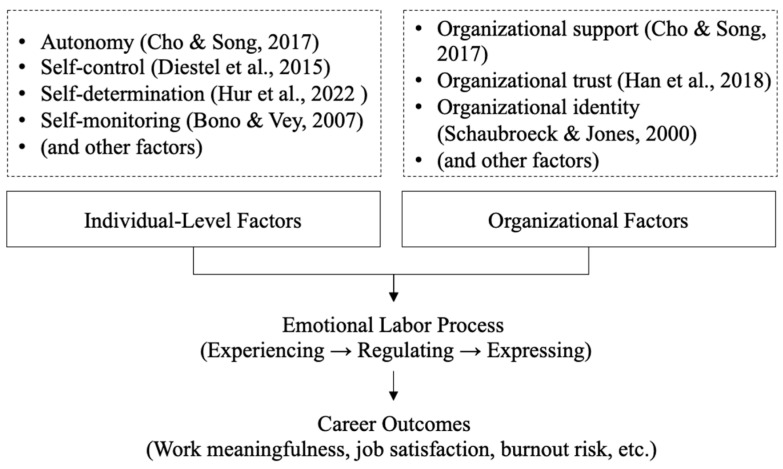
Individual- and organizational-level determinants of emotional labor (Cho & Song, 2017; Diestel et al., 2015; Hur et al., 2022; Bono & Vey, 2007; Han et al., 2018; Schaubroeck & Jones, 2000).

Prior studies have examined how both individual and organizational factors shape emotional labor in human services (see Figure 1, Bono & Vey, 2007; Cho & Song, 2017; Diestel et al., 2015; Grandey et al., 2015; Han et al., 2018; Hur et al., 2022; Schaubroeck & Jones, 2000). However, they are often treated as buffers against harm rather than catalysts for growth. Much of the research remains rooted in a compliance-based view—seeing emotional labor as a burden to be endured rather than a process to be understood (Humphrey et al., 2015). Moreover, emotional labor remains deeply gendered, yet the agentic dimension of this process remains underexplored in the existing literature. Women are expected to perform emotional work as part of their professional identity, but the effort is often invisible, undervalued, or misrecognized (Nguyen & Stinglhamber, 2020). While organizational interventions exist, they tend to overlook how gender norms shape emotional expectations, regulation strategies, and career outcomes. Specifically, prior research rarely examines how women actively negotiate these norms, including resisting prescriptive emotional display rules, redefining professional identity, or leveraging emotional skills for career advancement, nor how these agentic strategies differ from men’s experiences. This study attempts to address these gaps and illustrates how agency operates within gendered emotional labor.

To move beyond a predominantly reactive view of emotional labor, this study reconceptualizes it as a dynamic process—one in which individuals experience, regulate, and express emotion in agentic ways (Parker et al., 2010). Drawing on a nationally representative, multi-source dataset of social workers and their organizations in China, we examine how emotional labor unfolds differently by gender and how professional efficacy functions as a key mechanism in transforming emotional strain into work meaningfulness. We also investigate how organizational structures—particularly internal governance and governmental support—moderate this emotional process. In doing so, we offer a gendered, agentic framework tested with nationally representative, multi-source data that specifies the efficacy pathway and its institutional contingencies, and we translate this into actionable guidance on internal governance and efficacy-building interventions to enhance work meaningfulness in human services.

## 2. Literature Review and Theoretical Framework

### 2.1. Emotional Labor in the Workplace

Early career theories largely assumed rational decision-making and downplayed emotion (Kidd, 1998). Subsequent work in sociology and organizational psychology recognized emotions as social and communicative phenomena. Hochschild’s *The Managed Heart* introduced emotional labor as the management of feeling and display according to employer rules (Hochschild, 1983; Wharton, 2009). This marked a shift from neglect to commodification: Organizations train and evaluate workers on emotional displays to improve service outcomes, often privileging efficiency while overlooking broader effects on private life, lifestyles, and well-being (Smith, 2014). In service and public-sector occupations, these standards shape interactions with clients and teams and press workers to align internal states with institutional norms.

As the most prominent feature of emotional labor, gender is an inevitable topic. Kemper’s (1978) social relational theory suggests that power and status differences between the genders bring out differences in people’s emotional experience. Women’s lower status and level of power result in negative feelings in their social interactions, whereas men are likely to report positive emotions because of their sense of security. The emotion-management strategies adopted by the two genders are subject to cultural gender schemas, specifically in terms of surface acting and deep acting (Hochschild, 1983). Compared to women, men tend to be experienced in covering up their true feelings, and consequently they are more likely to adopt surface acting than women are (Simon & Nath, 2004). In regard to expressing emotion, men’s and women’s patterns of expression are shaped differently by gender expectations and the social division of labor. Gender socialization theory holds that the subtle encouragement from socialization agents enhances gender role stereotypes, such as “women are the masters of emotion”. Parsons’ functional theory about gender differences argues that the social division of labor requires men and women to specialize in different rules for their respective emotional roles (Simon & Nath, 2004).

Despite a decline in gender segregation and the encouragement of a diversity-friendly workforce, most people simultaneously expect men and women to pursue their “natural” jobs. The professions dominated by women, such as nurses, teachers, and maternity matrons, are regarded as extensions of women’s parenting role, and consequently their emotional efforts in those professions are taken for granted (McPhail, 2004). Most emotional work in human services organization requires vis-a-vis contact with the public, women are expected to behave with gentleness, approachability, and forbearance, and men are expected to be efficient organizers and problem solvers (Husso & Hirvonen, 2012). Emotional labor appears to draw upon females’ natural skills, and the research suggests that emotional labor in the social service sector runs the risk of perpetuating gender inequality (Casey et al., 2025; Y. Liu & Huang, 2025; Weinberg, 2021; Wingfield, 2021).

However, although a large number of studies have repeatedly verified the dark side of emotional labor, they tend to overlook not only the agentic efforts of individuals—especially female workers—in resisting and adapting to gendered role expectations (Humphrey et al., 2015), but also the broader organizational reflections on emotional governance. Against this backdrop, we attempt to reconceptualize the process of emotional labor, shifting the focus to emotional transformation and professional growth, and highlighting the constructive agency of emotional laborers in the workplace. This agentic perspective captures a dimension of emotional labor largely absent from prevailing accounts and opens up possibilities for constructing women’s subjectivity and realizing the “bright side” of their work within gendered occupational contexts.

### 2.2. Construction of an Emotional Labor Model That Considers Gender Differences

In regard to emotion management, Wharton (2009) introduced research discussing individuals’ experiences, regulation, and expression of their emotions. Briner and Totterdell (2002) described workplace emotions through three dimensions: emotional experience, emotional expression, and emotional regulation. Through expression, reception, and interpretation, emotions shape relationships, decision-making, and dynamic processes in professional contexts, even becoming a vital medium for individual growth and organizational change (Kidd, 2004).

In an active manage process, emotional laborers invest their abilities and professional skills in order to adjust, digest, and resist the emotional discomfort caused by role ambiguity. In the end, emotional laborers realize their job performance and self-achievement by transforming negative emotions into a service motivation. Therefore, we conceptualize role ambiguity—a major source of emotional stress in the workplace—as the primary form of emotional experience (Peiro et al., 2001), define emotional regulation as professional efficacy (Maslach et al., 1997), and interpret work meaningfulness as the expressive outcome of this emotional process (Chalofsky, 2003).

To further theorize the dynamic transformation between emotional experience and work outcomes, we draw on the proactive motivation model (Parker et al., 2010). This model explains how individuals initiate and sustain proactive behaviors in the workplace, comprising a motivational process that moves from “can do” (self-efficacy), through “reason to” (meaning and purpose), to “energized to” (active striving). By integrating this model with the emotional labor process, we conceptualize emotional regulation and expression not as passive adaptation, but as a proactive journey of self-mobilization that connects internal struggle with external achievement.

#### 2.2.1. Role Ambiguity and Work Meaningfulness

Role ambiguity refers to a lack of understanding of one’s job responsibilities and the expectations of one’s job performance (House & Rizzo, 1972). Individuals can lack clarity in their role cognition, and this can lead them to be confused, due to internal or external factors. Together with other similar concepts, such as role conflict and role overload, role ambiguity is a primary source of stress that has been proved to be positively related to personal burnout and negatively related to work meaningfulness (Peiro et al., 2001). Work meaningfulness refers to employees’ perception of the value and usefulness of their work, reflecting the positive experience of developing and expressing themselves in service roles. Emotional experiences at work are shaped by personal characteristics, emotional norms, and environmental interactions. When actual emotions conflict with prescribed display rules, emotional disorder may arise, leading to cognitive impairment and role ambiguity (Kwak et al., 2018). Maintaining emotional congruence and clear role boundaries can enhance role clarity and promote positive emotional expression.

Over the past few decades, the continuous organizational changes in the human services sector in terms of work environment have generated substantial levels of role ambiguity (Verlinden et al., 2022). Uncertainty in their work environment affects the extent to which individuals clearly understand their job responsibilities, job expectations, and organizational goals. Meanwhile, the “Smile Service” that requires emotional work also elicits a disconnection between workers’ selves and their roles and leads to ambiguity toward those roles (Polletta & Tufail, 2016), especially for emotion workers who adjust across multiple working conditions. Having constant role ambiguity and emotional burdens leads to professional burnout, emotional exhaustion, and value frustration and a reduced sense of achievement and meaningfulness in their work. However, another possibility lies in the nature of human services work itself: it often involves deep emotional engagement with vulnerable populations, making emotional labor not only a requirement but also a potential source of professional meaning and personal well-being.

#### 2.2.2. The Transforming Role of Professional Efficacy as a Proactive Force

Professional efficacy refers to individuals’ self-evaluation of their competence, productivity, and capacity to achieve professional goals (Maslach et al., 1997). Within the proactive motivation framework (Parker et al., 2010), efficacy beliefs play a pivotal role in determining whether negative emotional experiences are perceived as insurmountable obstacles or as signals for constructive change. When individuals maintain a strong sense of professional efficacy, they are more likely to engage in emotional regulation strategies that reframe strain as a source of purpose and opportunity, thereby initiating the transformation from emotional challenge to professional growth. In this way, professional efficacy functions not merely as a buffer against burnout, but as a catalyst for the agentic process through which emotional labor becomes a vehicle for meaning-making and identity consolidation.

Role ambiguity usually predicts that an ambiguous sense of achievement and efficacy will be a common source of stress, whereas professional efficacy can be predicted by a sense of command, autonomy, competence, and value (Chaudhary, 2022). Professional efficacy is a source of cognitive motivation that affects their way of affective adjustment and beliefs about job capability, and consequently helps to form cognitive assessments from emotional experiences (Strobel et al., 2021). By integrating professional efficacy into the proactive motivation framework, it may transform negative emotional triggers into opportunities for proactive action, which hinges on the individual’s belief in their capacity to manage and regulate emotional states effectively. A high level of professional efficacy may alleviate the person’s emotional burden and help to generate a perception of value and meaningfulness. In contrast, a low level of professional efficacy and a highly negative self-evaluation will lead workers into a vortex of emotions and self-deprecation. Therefore, we proposed the following:

**H1.**  *Professional efficacy (regulating) will play a mediating role in the relationship between role ambiguity (experiencing) and work meaningfulness (expressing).*

#### 2.2.3. Gender Differences in the Model Construction

Gender is an element infused within organizational structure, and it largely defines how emotional laborers feel about their environment, thus reshaping men’s and women’s identity and constructing their work meaningfulness through their emotion management (Cain, 2017). Knowing the different emotional process between the two genders helps us to learn about humanistic and delicate management practices in the work context.

Regarding the experiencing of emotion, women seem to face a greater emotional burden and role ambiguity than men do. Female hospice workers report that their male coworkers clearly have exceptional boundaries with patients, whereas they themselves cannot remain emotionally detached (Cain, 2017). Male nurses are also thought to lack caring skills, including empathy, warmth, and emotional support (Husso & Hirvonen, 2012). This may imply that women are more frequently confronted with negative emotional triggers that undermine their readiness-to-act state (Parker et al., 2010). In regard to regulating and expressing emotions, women are said to employ a wider variety of strategies, such as rumination, reappraisal, acceptance, distraction, and seeking social support, in managing their emotions (Nolen-Hoeksema, 2012), which strengthens their motivation and allows them to reframe role ambiguity into opportunities for growth (Parker et al., 2010). With respect to expressing emotions, women seem to have stronger autonomous motivations and higher efficacy in emotional processing than men do. A survey of females showed that their positiveness was more related to their interests and satisfaction and was independent of internal pressures and external constraints, whereas the males’ positiveness was not (Watt et al., 2013). Men are less likely than women to use emotional strategies, follow performance rules, or meet emotional expectations, which constrains their capacity to transform emotional experiences into work meaningfulness.

Therefore, we hypothesize that women and men follow different gendered agentic pathways of proactive emotional labor: women are more likely to engage in extended and transformative pathways that convert negative emotional experiences into meaningful expression through regulation.

**H2.**  *Gender differences in the effects of the emotional labor model will be explained by male–female differences in role ambiguity (experiencing) and professional efficacy (regulating) with work meaningfulness (expressing)*.

### 2.3. The Moderating Effect of the Institutional Environment

Research has consistently shown that organizational support plays a crucial role in shaping employees’ experiences and outcomes related to emotional labor. Institutional environment, encompassing both internal mechanisms and external frameworks, has been highlighted as a significant factor in mitigating the emotional costs of work and enhancing job satisfaction (Cho & Song, 2017; Schaubroeck & Jones, 2000). Building on these insights, we will materialize organizational governance into two dimensions: internal governance effectiveness and governmental support.

Organizational governance is frequently viewed through board processes, adherence to rules and regulations, formalized structures, and systems of control. The effectiveness of organizational governance affects the efficacy of members and the service level of agencies, which are characterized by accountability, transparency, fairness, and responsibility. Good governance means that organizational mechanisms, processes, and procedures are effective and that leadership decisions are consistent and of high quality. This ensures that roles in the workplace will be clearly defined and hidden emotional costs will be reduced (Hattke et al., 2020). By contrast, good governance can develop the organization’s capability to be effective, thus ensuring its internal vitality and positive external image. Furthermore, effective organizational governance can promote the values of the whole organization. Therefore, we proposed the following:

**H3.**  *Internal governance effectiveness will play a moderating role in the emotional labor model.*

In practice, human services providers in China generally suffer from a shortage of funds and a lack of motivation. Due to China’s unique institutional structure, the government has been given the right to intervene in human services organizations and human services providers, especially for those in the area of public education and medical care. The government is accustomed to providing human, financial, and material support generously for start-ups, and this support may offer organizations direct resource supplements and development opportunities for developing workers’ professional skills. However, managerial autonomy and service professionalism are somewhat limited when it comes to compliance with government regulations, and these limitations may trigger a decrease in employees’ professional efficacy. Apart from that dynamic, human services providers’ lower reward-to-reward ratio and social identity can reduce their level of occupational efficacy and personal fulfillment (Yan, 2022). Therefore, we proposed:

**H4.**  *Governmental support will play a moderating role in the emotional labor model.*

**Figure 2:**
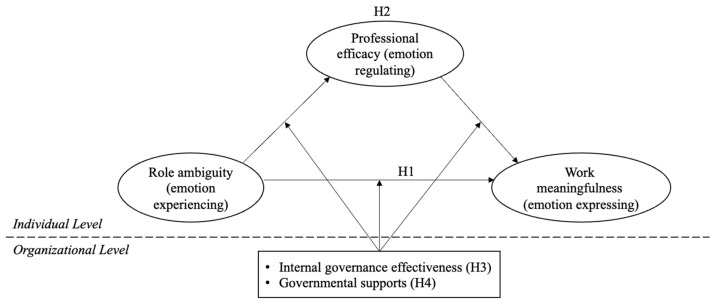
Conceptual framework.

Based on the above four hypotheses, we constructed the conceptual framework shown in Figure 2.

## 3. Methods

### 3.1. Sample and Procedures

To test our hypotheses, we utilized data from the first round of the China Social Work Longitudinal Study (CSWLS). Recognized as China’s first large-scale ongoing survey on social work development, the CSWLS adopted a multistage random sampling strategy across 56 cities and underwent four rounds of quality control to ensure data reliability. This study draws on multi-source data, comprising responses from 979 social work agencies and 5965 matched social workers. Specifically, 993 questionnaires were distributed to organizations (with 979 valid responses, 98.59% effective rate) and 6785 to social workers (with 6776 valid responses, 99.87% effective rate). The dataset allows for the integration of individual-level variables (e.g., gender, efficacy, role ambiguity) with organizational-level information (e.g., internal governance, governmental support), enabling us to examine institutional moderation effects within a cross-sectional design. Among the individual respondents, 79% were female, with an average age of 30.44 years; 24% were members of the Communist Party; 53.4% held a bachelor’s degree, and 60.5% had at least an assistant social work license. On the organizational side, 77.46% of agencies were overseen by civil affairs departments. The average age of organizations was 4.99 years (ranging up to 30), with an average staff size of 27.92 (ranging from 2 to 800). Approximately 73.90% provided comprehensive services. Approximately 73.90% provided comprehensive services. To ensure data validity, only formally registered organizations with at least one year of operation and staff with a minimum of three months’ tenure were included, while unregistered agencies, temporary workers, and incomplete questionnaires were excluded. With respect to data quality, questionnaires with more than 20% missing responses were removed from the dataset. For the remaining cases, given that the overall rate of missing data was below 5%, we adopted listwise deletion to handle missing values (C. Liu et al., 2020). Table 1 reports the descriptive statistics of the individual and organizational samples.

### 3.2. Measurements

**Three Research Variables**. To ensure construct validity for the three core latent variables—role ambiguity (RA), professional efficacy (PE), and work meaningfulness (WM)—we derived survey items from the existing literature. We utilized the Chinese version of the role ambiguity scale (Rizzo et al., 1970), employing a five-point Likert scale from 1 (strongly disagree) to 5 (strongly agree). To facilitate interpretation, we reversed the scoring of all items so that lower scores indicated lower role ambiguity. The overall Cronbach’s alpha for this scale was 0.803.

Next, we evaluated professional efficacy with an eight-item subscale from the MBI-GS (Maslach et al., 1997), a self-report questionnaire consisting of three subscales: five items on exhaustion (EX), five on cynicism (CY), and eight on professional efficacy (PE). All items pertain to burnout in the workplace. High scores in EX and CY combined with low PE indicate potential burnout. This questionnaire used a seven-point Likert scale, ranging from 0 (never) to 6 (daily), with a Cronbach’s alpha coefficient of 0.932.

Lastly, we assessed work meaningfulness—reflecting how meaningful individuals find their work—using four items adapted from Arnold et al. (2007). Example items included “I find real pleasure in my work” and “I am passionate about my work.” Respondents rated these on a five-point scale from 1 (strongly disagree) to 5 (strongly agree), with the scale yielding a Cronbach’s alpha coefficient of 0.826.

**Moderators**. In alignment with our research objectives, we included two organizational-level variables (internal governance effectiveness and governmental support) to explore potential moderating effects of institutional environments. Internal governance effectiveness (IGE) was measured with seven items from the CSWLS organizational survey (Ye & He, 2021), assessing the board’s effectiveness in areas such as senior manager selection, resource mobilization, marketing, mission development, and financial oversight (Cronbach’s α = 0.95; χ^2^(21) = 5963.01, SRMR = 0.04, CFI = 0.92, TLI = 0.96, RMSEA = 0.10). We summed the items and split the sample into two groups based on the mean: high IGE (*n* = 472) and low IGE (*n* = 498). Governmental support (GS) was evaluated using six items: funding for purchases, supplementary funds, site support, manpower support, technical assistance, and policy facilitation (Cronbach’s α = 0.76; χ^2^(15) = 671.01, SRMR = 0.05, CFI = 0.92, TLI = 0.95, RMSEA = 0.09). Similar to IGE, we summed these items and divided the sample into two groups based on the mean: high GS (*n* = 594) and low GS (*n* = 376).

**Measures for Covariates**. To align with prior research, we controlled for participants’ gender, age, years of experience (tenure), and educational background in social work in our models.

## 4. Results

The Results Section consists of three primary components: (a) confirmation of the measurement model, (b) structural equation modeling that includes mediating effects from a gender perspective, and (c) analysis of moderating effects. This study explored how professional efficacy functions within the relationship between role ambiguity and work meaningfulness among human services providers. We then compared the effect sizes of male and female emotional models to further clarify variations among human services providers. Finally, we assessed the moderating effects of two institutional variables: internal governance effectiveness and governmental support.

### 4.1. Measurement Model Confirmation

To control for statistical bias, we initially conducted Harman’s one-factor test. An exploratory factor analysis revealed that the first unrotated factor, with an eigenvalue greater than one, accounted for 41.291% of the total variance, which is below the 50% threshold recommended by Podsakoff et al. (2003). While these findings do not eliminate the possibility of common method variance (CMV), they suggest that CMV was not substantial enough to influence the results of this study.

Next, we evaluated the measurement model’s adequacy, which is crucial for ensuring the reliability and validity of the scales. We performed confirmatory factor analyses using maximum likelihood estimation with Amos 24 software to assess reliability, convergent validity, and discriminant validity. The measurement model comprising three latent variables (referred to as Model in Table 2) exhibited a good fit (*χ*^2^/*df* = 2.816; CFI = 0.982; RFI rho 1 = 0.959; RMSEA = 0.043). All constructs achieved composite reliability coefficients exceeding the acceptable threshold of 0.7, indicating strong internal consistency. For convergent validity, we utilized criteria such as average variance extracted (AVE > 0.5; CR > AVE > MSV > ASV) (Hair et al., 2010). All variables met the standards for convergent validity, as detailed in Table 2. Following Fornell and Larcker (1981), we assessed discriminant validity by comparing the square roots of the AVE with inter-construct correlations.

### 4.2. Structural Model

We first tested an initial structural model examining the impact of role ambiguity on work meaningfulness among human services providers, without including the mediator of professional efficacy. The initial model fit (Model 0) was satisfactory (*χ*^2^/*df* = 3.023; CFI = 0.989; RFI rho 1 = 0.966; RMSEA = 0.046), showing a significant positive relationship between role ambiguity and work meaningfulness (*β* = 0.45 ***, *R*^2^ = 0.20).

#### 4.2.1. Basic Theoretical Model

**Figure 3:**
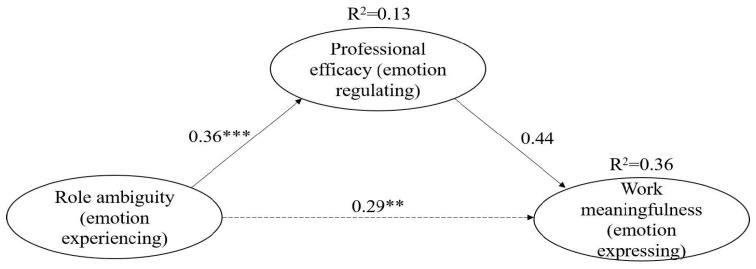
The results of the work meaningfulness model. ** *p* < 0.01, *** *p* < 0.001.

A theory-based structural model was developed to explore how role ambiguity affects work meaningfulness through professional efficacy, labeled Model 1. This model also demonstrated a good fit (*χ*^2^/*df* = 2.840; CFI = 0.982; RFI rho 1 = 0.964; RMSEA = 0.044). In Model 1, role ambiguity was significantly and positively linked to professional efficacy (*β* = 0.36 ***, *R*^2^ = 0.13) and to work meaningfulness (*β* = 0.44 ***). Notably, the relationship between role ambiguity and work meaningfulness became less pronounced (decreasing from *β* = 0.45 *** in Model 0 to *β* = 0.29 ** in Model 1), indicating a potential partial mediating effect. Together, role ambiguity and professional efficacy accounted for 36% of the variance in work meaningfulness, as illustrated in Figure 3.

**Mediating Effects**. The reduction in significance of the direct effect of role ambiguity on work meaningfulness (from *β* = 0.45 *** in Model 0 to *β* = 0.29 ** in Model 1) prompted us to utilize bootstrapping in AMOS 24.0 to assess potential mediating effects. Using the recommended 95% confidence intervals (bias-corrected percentile method) and 2000 bootstrap samples (Hayes & Preacher, 2010), we found that professional efficacy served as a partial mediator, moderating the impact of role ambiguity on work meaningfulness. The indirect effect (0.151 ***) was statistically significant, as was the direct effect (0.275 ***) in Model 1, thereby supporting Hypothesis 1. In other words, professional efficacy partly explains why social workers who feel clearer about their roles also report a stronger sense of meaningfulness in their work.

#### 4.2.2. Theoretical Model from the Perspective of Gender

**Figure 4:**
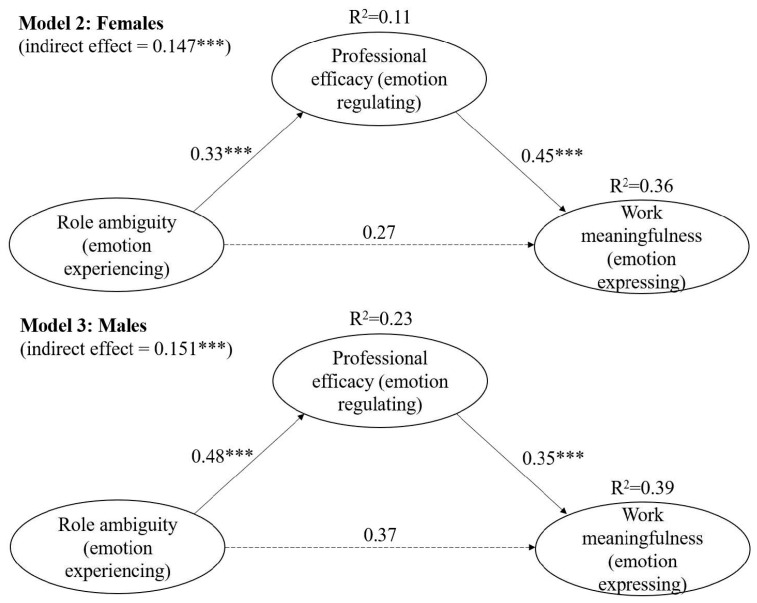
The compared models of work meaningfulness from a gender perspective. *** *p* < 0.001.

We compared theory-based structural models for female (Model 2) and male (Model 3) service providers (Figure 4, Table 3). Both models showed a good fit. Model 2 (female-emotion model) fit the data well (*χ*^2^/*df* = 2.448; CFI = 0.982; RFI = 0.960; RMSEA = 0.044). Role ambiguity was positively related to professional efficacy (*β* = 0.33 ***, *R*^2^ = 0.11) and work meaningfulness (*β* = 0.45 ***), explaining 36% of the variance. Model 3 (male-emotion model) also fit adequately (*χ*^2^/*df* = 1.909; CFI = 0.906; RFI = 0.890; RMSEA = 0.066). For males, role ambiguity showed stronger associations with professional efficacy (*β* = 0.48 ***, *R*^2^ = 0.23) and work meaningfulness (*β* = 0.35 ***), accounting for 39% of the variance.

Mediation analyses using 2000 bootstrap samples in AMOS 24.0 (bias-corrected percentile method, 95% CI, Hayes & Preacher, 2010) indicated partial mediation by professional efficacy in both groups (Table 4). Among females, the direct effect of role ambiguity on work meaningfulness decreased from *β* = 0.42 *** to *β* = 0.27 **, with significant indirect (0.115 ***), direct (0.266 ***), and total (0.413 ***) effects. For males, the direct effect dropped from *β* = 0.54 *** to *β* = 0.37 **, with indirect (0.151 ***), direct (0.333 ***), and total (0.484 ***) effects all being significant. Thus, H2 was supported.

Overall, the mediation pattern was consistent, but the male model exhibited larger direct and total effects, whereas indirect effects were similar across genders. This suggests that while professional efficacy plays a comparable mediating role, the broader impact of role clarity on meaningful work is stronger among men, underscoring the importance of considering gender when addressing role-related interventions.

### 4.3. Moderating Effects of Potential Boundary Conditions

**Figure 5:**
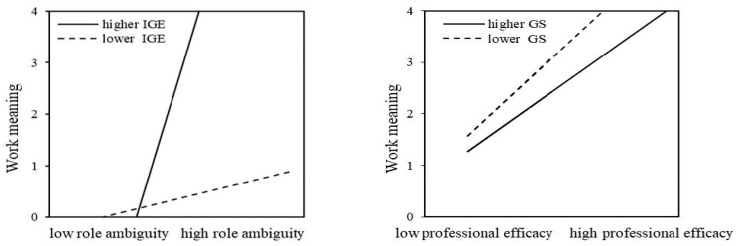
Moderation effects.

We then examined the moderating effects of two organizational-level variables—internal governance effectiveness (IGE) and governmental support (GS)—to further explore the conditions under which role ambiguity affects work meaningfulness for human services providers (Figure 5). A multiple-group analysis was performed using Amos 24 software to assess these moderating effects.

First, we assessed internal governance effectiveness as a moderator in the female (Model 2) and male (Model 3) models, both of which demonstrated a good fit (*χ*^2^*/df* = 1.984; CFI = 0.982; RFI rho 1 = 0.946; RMSEA = 0.025 for Model 2; *χ*^2^*/df* = 1.855; CFI = 0.942; RFI rho 1 = 0.847; RMSEA = 0.046 for Model 3). The results indicated that IGE moderated the relationship between role ambiguity and work meaningfulness only for male human services providers, as indicated by the *z*-score (2.946 ***). Specifically, the positive relationship between role ambiguity and work meaningfulness was more than one-tenth stronger among male service providers in high-IGE organizations (*β* = 0.566 ***) compared to those in low-IGE organizations (*β* = 0.042). Thus, Hypothesis 3 was partially supported.

Next, we evaluated governmental support as the second moderator in both models, which again showed a good fit (*χ*^2^/*df* = 2.044; CFI = 0.971; RFI rho 1 = 0.945; RMSEA = 0.026 for Model 2; *χ*^2^/*df* = 1.828; CFI = 0.945; RFI rho 1 = 0.850; RMSEA = 0.045 for Model 3). We found that GS moderated the relationship between professional efficacy and work meaningfulness specifically for female human services providers, as indicated by the *z*-score (1.336 *). The positive impact of professional efficacy on work meaningfulness was significantly stronger among female service providers in low-GS organizations (*β* = 0.268 ***) compared to those in high-GS organizations (*β* = 0.206). Thus, Hypothesis 4 was partially supported. These results suggest that governmental and organizational contexts play a crucial role in shaping the effects of role ambiguity and professional efficacy, highlighting the need for tailored strategies that account for gendered differences in work meaningfulness.

## 5. Discussion

### 5.1. Emotional Labor and Gendered Vocational Insights at the Theoretical Level

We found that the effect of men’s experiencing of emotions on their expression of emotions was stronger than that in women, but there was no significant difference between men and women in their regulation of their emotions. Our findings indicated that female human services providers need more emotional transitions via professional efficacy when confronted with an emotional burden. By contrast, the emotional processing of males (the path from experiencing to expressing) is more direct and concise.

Our conclusions about the gender difference in emotional processing is consistent with previous studies (Nolen-Hoeksema, 2012). Women showed better emotional awareness when confronted by role ambiguity, and they gave more attention to and had a better understanding of their emotions. Compared with men, women are more willing to change positively, such as by exercising professional efficacy to help them adapt to their work environment and job requirements. We found that there also is a bright side of emotional labor, while disputes continue: women’s agentic capacity of regulation through their professional efficacy can bring them enhanced self-achievement and work meaningfulness.

Grounded in the theory of emotional labor, being full of the emotions that norms and social structures require has been internalized into gender awareness and embedded in the female habitus, and that awareness determines their practices and perception in a persistent tendency system in the female-dominated workplace. Meanwhile, the proactive motivation perspective helps to explain how women high in professional efficacy stimulate their self-awareness through the perspective of the Other, thus helping them to agentically regulate their emotions and keeping them from sinking into role ambiguity. Women take their role clarity and the actual self that their professional efficacy prompts and use them to inspire their underlying motivation and seek a more comprehensive self-concept in their emotional interactions.

Their entire emotional labor process is agentic and reflective, such that their emotion is self-directed, not just socialized. From a critical perspective, the expansion of emotional services in the human services sector creates a potential means of control and alienation for the employees that emotional labor is imposed upon (Krstić et al., 2025; Wharton, 2009). Women’s emotional skills are shaped by their social status and gender cultural expectations and are commodified by public demands. Their emotional efforts are always regarded as natural skills to be provided additionally and deservedly. It is this belief—that emotional labor is simply an extension of their domestic role—that assigns women emotional responsibility and diminished status (Husso & Hirvonen, 2012). In a positive sense, however, their stronger capacity for emotional regulation skills simultaneously offers a possibility for their self-liberation. Women not only exercise emotional reflexivity to help themselves perceive and modify the difference between the self and the workplace, but they also take advantage of expert knowledge to transform themselves positively through internal conversations (Archer, 2003). Women begin the reflective process as individualists and rationalists, clarify it through self-awareness and with a constructed role identity, and ultimately they establish a relationship between emotional regulation and meaningful expression (Burkitt, 2012).

This finding provides us insights into women’s work practices and organizational governance. Although gender inequality pervades the emotional labor professions, it also guides the way females reflect and respond, and how they negotiate their roles in interactions (Parker et al., 2010; Peiro et al., 2001). Women who use professional efficacy have learned to counteract their emotional negativity and enhance their work meaningfulness, and these benefits provide pathways that contribute to feminist practices (Parker et al., 2010; Strobel et al., 2021). The positive outcomes displayed in cases not only implicate emotional laborers to evolve from being passive, “emotion-exploited” individuals to being agentic “emotional managers,” they also allow them to maintain work meaningfulness across time and space (Polletta & Tufail, 2016; Mei, 2020).

Interestingly, we also found this dynamic to be more pronounced under insufficient government support—a result that we tentatively interpret as being associated with the Chinese context. In China, the government is accustomed to providing human, financial, and material support through speedy administrative means in human services agencies’ foundation stage. Governmental support implies an authorized recognition, potential for development, and professional worth. The emotional experience gap between the lack of acknowledgment symbolized by a shortage of governmental support and the strong sense of the inherent value of emotional labor may increase the likelihood of women’s inward-seeking and self-reflexive emotion regulation—women might search for other ways to express their value, to enhance their sense of self-worth, and to fulfill their lost work meaningfulness (Lin, 2019). Humphrey et al. (2015)’s research supported our findings. When laborers lack adequate support and resources for too long, their emotional labor can have a significant and negative impact on their well-being and performance over time (Delgado et al., 2017). Nevertheless, given the cross-sectional nature of our data, these interpretations should be viewed with caution.

In contrast to women’s unexpected performance in situations of low government support, we also found that the level of transformation from role conflict to work meaningfulness was much higher (10 times) for men in high-internal-effectiveness organizations than it was for men in low-internal-effectiveness organizations, which deepens our understanding of the association between gender discourse and emotional labor in modern organizational governance. As was stated previously, gender socialization shapes gender role stereotypes, and these stereotypes affect the gender-normative behaviors of both women and men. Gender role norms form men’s goal-oriented rational characteristics while also shaping women’s emotional characteristics (Gilligan, 1993). Organizations with high internal governance effectiveness are implied to have efficient processes, effective mechanisms, impartial procedures, and clear responsibilities, all of which allow men to better identify rationalized arrangements and deal with less role ambiguity when facing emotional burdens. High IGE may also reduce burnout caused by experiencing emotions and consequently can shorten the processing path of men’s emotional rumination. Meanwhile, the masculinity implicit in the organizational rationality of purchasing efficiency also affects organizational discourse and management practices, thus providing a more supportive environment for men’s rationality (Ross-Smith & Kornberger, 2004). These findings offer theoretical and practical information for the organizational governance of both genders of emotional laborers.

### 5.2. Humanistic Management at the Practical Level

Building upon the proactive motivation model, which highlights the individual’s role in managing emotional labor, we argue that it is thus important to consider the organizational role in creating environments that support employees’ emotional well-being. Proactive emotional labor requires not only a person-centered focus on individual efforts to handle emotional challenges but also humanistic management that emphasizes systemic organizational adjustments with an employee-centered approach. On one hand, humanistic management acknowledges emotional effort. Emotions and their worth are an important part of emotional work and are taken seriously, and this may be important for human services providers in their efforts to realize their work meaningfulness (Berry et al., 2024). On the other hand, humanistic management ensures gender-differentiated management—a holistic care-based approach that is based on compassion and empathy humanizes personnel management and values individuals’ dignity and identity, which is essential for emotional labor with a distinctive gender feature (Slater, 2021). Therefore, we suggest separate governance focuses for men and for women, tailored to meet their respective gender-specific governance needs.

First, our findings validate the superiority, importance, and inevitability of effective organizational governance. Organizational leaders and policymakers should strengthen transparent decision-making procedures, clarify role expectations, and establish fair channels of recognition and reward, thereby reducing gendered role ambiguity and promoting employees’ well-being. At the same time, given the particular ways in which our effects were displayed among men, we suggest that improving organizational effectiveness can further enhance men’s positive emotional experiences. Human services organizations can optimize organizational processes, shape their organizational culture, maintain openness and transparency, and make consistent decisions to ensure role clarity and to continually improve their internal governance. The hierarchy, specialization, formalization, standardization, centralization, and flexibility of an organization can ensure efficient organizational operations (Melé, 2016). Future cross-cultural and longitudinal studies could test the extent to which these dynamics generalize across different institutional environments.

Second, our findings confirmed that emotional laborers who are engaged in human services may need greater recognition of the value of their emotional labor and the meaningfulness of their work, especially for female human services providers. Basing our thinking on proactive motivation theory, we recommend that organizational managers value their workers’ emotional worth, work meaningfulness, and self-realization in the workplace. Those valuations can be enhanced in two specific ways. On the one hand, mastering strategies for expressing emotion and enhancing emotional skills can help human service workers to transform negative emotions into positive ones. Therefore, we recommend that emotional laborers learn to recognize their own emotional labor, actively monitor whether they are over-consuming emotional energy in daily work, and treat emotional labor as a cultivable ability. Practitioners should be reminded to regard emotional labor as a skill to be developed, to find balance in role conflicts, and to avoid long-term burnout. We also advise organizations to develop and train their female emotional laborers to clearly perceive their professional behavior and gradually build their confidence in reaching their professional goals. It is also important to foster job competency and professional efficacy by participatory management and greater opportunities for professional development. Furthermore, organizations should set rules about emotions on the basis of humanism instead of mandating strictly enforced rules, and they should allow human services providers and clients to independently negotiate their worker–client relationships through organized emotional care (Lopez, 2006).

On the other hand, organizational managers should seek to enhance the image and visibility of the organization in an effort to strengthen the professional identification of the emotional laborers. Being perceived positively as a professional has important implications for staff recruitment, public acceptance, the vitality of the profession, and individuals’ sense of pride and identity (Legood et al., 2016). The low status and economic devaluations in some human services professions make it necessary to develop strategies and construct meaning from their work experience, within constraints, and greatly influence emotional laborers’ professional identification and the meaningfulness of their work. Meanwhile, within the organization, managers are advised to help emotional laborers maintain their self-care, minimize the issues of low wages, and acknowledge the qualities they possess (Yan, 2022).

### 5.3. Limitations

It is important to acknowledge several limitations of this study. Firstly, we could not address the issue of endogeneity, which is often present in cross-sectional research, making it impossible to definitively conclude that the observed relationships are causal. This limitation also constrains the interpretation of our mediation and moderation analyses, as cross-sectional data cannot fully capture temporal ordering or dynamic processes. However, the use of nationally representative, multi-source data—including matched organizational- and individual-level information—helps to enhance the robustness and contextual depth of our findings. We suggest that future research could include longitudinal multilevel designs or experimental approaches (e.g., training interventions for social sector emotion workers to manage negative emotions) to provide stronger causal evidence. Moreover, another limitation concerns the possibility of omitted variables and measurement constraints. In measuring organizational-level moderators (internal governance effectiveness and governmental support), we dichotomized continuous indicators into “high” and “low” groups for theoretical clarity and interpretability. This choice, however, reduces variability and may entail information loss. Future research could instead treat these moderators as continuous variables and test interaction terms directly. Future studies could also explicitly consider organizational-level factors, such as organizational climate and leadership style, which may shape how emotional labor is enacted and experienced. Adopting cross-level modeling approaches would help to capture a broader range of influencing factors and provide deeper insights into how emotional labor and professional agency operate across different levels of analysis.

## Figures and Tables

**Table 1:** Descriptive statistics of individual and organizational samples.

	N (%)/Mean (SD)
**Individual respondents (N = 5965)**	
Gender	
Male (%)	21.00
Female (%)	79.00
Age (mean)	30.44
Communist Party member (%)	24.00
Education	
High school or below (%)	10.30
Junior college/Associate’s degree (%)	28.45
Bachelor’s degree (%)	53.40
Master’s degree or above (%)	7.85
Assistant social work license or above (%)	60.50
**Organizational sample (N = 979)**	
Overseen by civil affairs department (%)	77.46
Organization age (mean)	4.99
Staff size (mean)	27.92
Functional orientation	
Comprehensive services (%)	73.90
Specialist services (%)	100

**Table 2:** The reliabilities, validities, and inter-correlations.

	CR	AVE	MSV	ASV	WM	PE	RA
Work Meaningfulness (WM)	0.857	0.600	0.293	0.246	0.774		
Professional Efficacy (PE)	0.927	0.615	0.293	0.213	0.541	0.784	
Role Ambiguity (RA)	0.827	0.546	0.200	0.167	0.447	0.366	0.739

**Table 3:** The fits of the structural models.

Models	Descriptive	*χ*^2^/*df*	GFI	CFI	RFI rho1	RMSEA
Model 1	Emotion Model	2.840	0.963	0.982	0.963	0.044
Model 2	Emotion Model—Female	2.448	0.965	0.982	0.960	0.044
Model 3	Emotion Model—Male	1.909	0.906	0.958	0.890	0.066

**Table 4:** Mediating effect of RA-PE-WM model.

	Point Estimate	Product of Coefficient		Bootstrap 2000 Times, 95% CI (Bias-Corrected Percentile Method)		
		SE	z	Lower	Upper	*p* Value
Direct effect	0.275	0.039	7.051	0.203	0.354	0.001
Indirect effect	0.151	0.021	7.190	0.113	0.194	0.001
Total effect	0.426	0.048	8.875	0.334	0.519	0.001

## Data Availability

The data used in this study are from the China Social Work Longitudinal Study (CSWLS). The dataset is not publicly available but can be obtained upon reasonable request by contacting the official CSWLS team via email at issw@mail.ecust.edu.cn.
